# Biological characterization and selection criteria of adjuvant chemotherapy for early breast cancer: experience from the Italian observational NEMESI study

**DOI:** 10.1186/1471-2407-12-216

**Published:** 2012-06-06

**Authors:** Matteo Clavarezza, Giorgio Mustacchi, Andrea Casadei Gardini, Lucia Del Mastro, Andrea De Matteis, Ferdinando Riccardi, Vincenzo Adamo, Enrico Aitini, Domenico Amoroso, Paolo Marchetti, Stefania Gori, Francesco Carrozza, Evaristo Maiello, Francesco Giotta, Davide Dondi, Marco Venturini

**Affiliations:** 1 Ospedale Sacro Cuore Don Calabria, Negrar, Verona, Italy; 2 Ospedale Civile, Centro Oncologico, Trieste, Italy; 3 IRST, Meldola, Forlì, Italy; 4 IRRCS A.O.U. San Martino IST, Genoa, Italy; 5 Istituto Nazionale Tumori, Naples, Italy; 6 AORN Cardarelli, Naples, Italy; 7 A.O. Policlinico G. Martino, Messina, Italy; 8 A.O. Carlo Poma, Mantova, Italy; 9 Ospedale Unico Versilia, Lido di Camaiore, Lucca, Italy; 10 A.O. S. Andrea, Rome, Italy; 11 Ospedale Santa Maria della Misericordia, Perugia, Italy; 12 Ospedale A. Cardarelli, Campobasso, Italy; 13 IRCCS Casa Sollievo della Sofferenza, San Giovanni Rotondo, Foggia, Italy; 14 IRCCS Oncologico, Bari, Italy; 15 Sanofi-Aventis, Milan, Italy; 16 Oncology Department, Ospedale Sacro Cuore Don Calabria Via Don A. Sempreboni 5, 37024, Negrar, Verona, Italy

## Abstract

**Background:**

International treatment guidelines recommend administration of adjuvant chemotherapy in early breast cancer based on clinical, prognostic and predictive parameters.

**Methods:**

An observational study (NEMESI) was conducted in 63 Italian oncology centres in patients with early breast cancer. Age, performance status, concomitant disease, menopausal status, histology, tumor dimension (pT), axillary lymph node status (pN), grading (G), estrogen and progesterone receptor (ER and PgR), proliferative index (ki67 or MIB-1), human epidermal growth factor receptor 2 (HER2) and type of adjuvant treatment were recorded. The primary objective of the study was to define parameters influencing the decision to prescribe adjuvant chemotherapy and the type of chemotherapy.

**Results:**

Data for 1894 patients were available. 69.0% postmenopausal, 67.0% pT1, 22.3% pTmic/pT1a/pT1b, 61.0% pN0, 48.7% luminal A, 18.1% luminal B, 16.1% HER2 positive, 8.7% triple negative, 8.4% unknown. 57.8% received adjuvant chemotherapy: 38.1% of luminal A, 67.3% luminal B, 88.2% HER2-positive, 97.6% triple negative. Regimens administered: 9.1% CMF-like, 48.8% anthracyclines, 38.4% anthracyclines plus taxanes, 3.7% taxanes alone. Increasing pT/pN and, marginally, HER2-positive were associated with the prescription of anthracyclines plus taxanes. Suboptimal schedules (CMF-like or AC/EC or FEC-75) were prescribed in 37.3% receiving chemotherapy, even in HER2-positive and triple negative disease (36.5% and 34.0%, respectively).

**Conclusions:**

This study showed an overprescription of adjuvant chemotherapy for early breast cancer, particularly referred to luminal A. pT, pN and, marginally, HER2 were the principal determinants for the choice of chemotherapy type. Suboptimal chemotherapy regimens were adopted in at least one third of HER2-positve and triple negative.

## Background

Breast cancer is the most common malignancy in women in Italy and its incidence is similar to the data from other tumor registry of other countries such as NORDCAN [1], SEER [2], UK [3]. Breast cancer incidence increased until 2000. From 2003 breast cancer incidence in USA is little decreasing for the reduced administration of hormonal replacement therapy [4,5], while in Italy from 2000 incidence is stable above all because of completed extension of mammographic screening [6-8]. In Italy breast cancer incidence vary according to geographic location. In fact, it is 115 cases per 100,000 women (45000 new cases each year), consisting of 29% of all new diagnosis of malignancies, with significant differences between North, Centre and South Italy: 123, 103 and 87 new cases are diagnosed each year per 100,000 women, respectively [9]. This difference is due to the different extension of mammographic screening and different risk factors for breast cancer, above all the different life-styles, with North Italy with a life-style more similar to North Europe and United States compared to South Italy.

Breast cancer causes more than 11,000 deaths each year in Italy, 24.2 cases per 100,000 women/year, with no relevant difference between North, Centre and South Italy (24.8, 21.1, 24.7 deaths for breast cancer per 100000 women/year), even if 5-year overall survival is between 85% and 87% in North and Centre Italy, compared to 81% of South Italy [9]. This difference is probably due to the different possibility to access to screening program [10].

Optimal management of patients with early breast cancer involving integrated treatments encompassing surgery, radiotherapy and systemic therapy had led, together to breast cancer screening, to mortality reduction [11]. Tumor stage, histological grading, estrogen and progesterone receptor (ER and PgR), proliferative index and human epidermal growth factor receptor 2 (HER2) aid in tumor evaluation and often dictate the choice of systemic treatment. Evidence-based international treatment guidelines including those of the NCCN (National Comprehensive Cancer Network) [12], the National Cancer Institute (NCI) [13] and the St. Gallen Consensus Report [14] recommend using adjuvant therapy for patients with early breast cancer. In particular adjuvant chemotherapy is not recommended for endocrine-sensitive breast cancer (ER-positive, low proliferative index and HER2 negative) with low recurrence risk. On the contrary, it is particularly indicated in at least one of the following biological conditions: ER-negative, HER2 positive, high proliferative index (>30%), grading 3. It remains unclear which is the role of adjuvant chemotherapy in endocrine sensitive breast cancer with intermediate recurrence risk (tumor diameter of 2 cm or more or node-positive from one to three lymph nodes), grading 2, proliferation index below 30%.

The aim of this retrospective observational study is to analyze factors influencing the choice for adjuvant chemotherapy, type of chemotherapy and schedules of chemotherapy in patients with early breast cancer in order to improve knowledge about the therapeutic management of early breast cancer in Italy and verify the adherence to international guidelines.

## Methods

### Study design

NEMESI is a retrospective observational study conducted at oncology unit throughout Northern, Central and Southern Italy. Institution types included IRCCS (Istituto di Ricovero e Cura a Carattere Scientifico), public hospital and private hospitals with oncology unit, public and private university hospitals. Centres were selected from the 386 censed centres (complex oncology units or simple oncology units with or without breast units) reported in the White Book of the Italian Association of Medical Oncology (AIOM 2006) in order to covering the whole spectrum of treatment of breast-cancer patients [15]. Centres were selected to represent Italian scenario on the basis of three criteria. The first was a geographic criteria: each area (North, Center and South-Islands) was to be proportional to the centres included in Libro Bianco (North Italy: 33 participating centres of 179 censed centres of Libro Bianco, which represent 52.4% of all centres of the study and 46.4% of all centres of Libro Bianco, respectively; Central Italy: 11 participating centres of 92 censed centres of Libro Bianco, which represent 17.5% of all centres of the study and 23.8% of all centres of Libro Bianco, respectively; Southern Italy and Islands: 19 participating centres of 108 of all centres of Libro Bianco, which means 30.1% of all centres of the study and 29.8% of all centres of Libro Bianco, respectively). The second selection criterion was that each type of Institution must to be rapresentated proportionally to Libro Bianco (7.5% of Private Hospital, 7.5% of IRCCS, 70% of Public or Private Hospital with Oncology Unit and 15% of public and Private University Hospital). The third criterion was to select centres with at least 120 new cases attending each oncologic centre in 2008 in order to ensure anonymity as required by Italian regulations governing observational studies (AIFA document, dated 30 March 2008).

Patients were eligible for the study if they met the following criteria: women, age ≥18 years; histological diagnosis of stage I, II or III breast cancer according to the tumor, node, metastasis (TNM) classification devised by the American Joint Committee on Cancer (AJCC) version VI [16]; undergone surgery; prescribed and received at least one cycle of adjuvant chemotherapy and/or prescribed adjuvant hormonal therapy; availability of data for the following parameters: tumor dimension (pT), axillary lymph node status (pN), grading (G), ER and PgR, HER2, and proliferative index (Ki67 or MIB-1). However, patients with lacking of one or more of these parameters were eligible and included in the analysis in order to obtain a real photograph of data available for the process choice of adjuvant chemotherapy. Candidates for adjuvant therapy with trastuzumab and/or radiotherapy on the residual breast or thoracic wall, regional supraclavear, internal breast lymph node stations were also eligible. Exclusion criteria were: use of neo-adjuvant therapy, diagnosis of metastatic breast cancer (stage IV) or in-situ carcinoma. Data were retrieved retrospectively by each site from the patients’ clinical records.

The protocol was reviewed by the independent ethics committee of the coordinating center. Notification of the study was also sent to the ethics committees of each participating center, as required by Italian regulations governing observational studies (AIFA document, dated 30 March 2008). The protocol complied with the Declaration of Helsinki.

### Sample size determination and data collection

Each center was requested to collect, by December 2009, data from a minimum of 10 and a maximum of 30 patients with breast cancer attending the center between 1 January 2008 and 30 June 2008. Each center had two particular requests in the selection of cases. First: data were collected by each center in a consecutive manner from a chronological point of view for patients attending oncologic units in the indicated period in order to exclude any selection bias. Second: each center had the request to collect data from at least 33% of patients receiving adjuvant chemotherapy. This particular inclusion criterion was necessary in order to obtain a sufficient number of patients receiving adjuvant chemotherapy in order to analyze, from a statistical point of view, parameters involving the choice to administer adjuvant chemotherapy. Data were collected on an electronic clinical report form and were submitted for automatic checks to assess completeness, correctness and internal coherence.

### Statistical analysis

Continuous variables were summarized using descriptive statistics, including number of subjects, mean, standard deviation, median, minimum, 25th percentile, 75th percentile and maximum. For categorical variables, summaries included counts of subjects and percentages. Multivariate logistic regression analysis was used to assess the relationship between clinical and demographic variables, choice of adjuvant chemotherapy, the regimen administered, and the following covariates: patient age, menopausal status, previous breast cancer history, ECOG performance status, surgery type, tumor stage, ER, PgR, HER2 and proliferative index. ER/PgR status and proliferative index were included in the model as categorical variables as well as continuous variables. A stepwise procedure was used with a significance level of p = 0.05 to retain variables in the model. Odds ratio (OR) estimates and their 95% confidence intervals (CIs) were also calculated. Two-sided 95% CIs for proportions and means are reported where applicable. The Statistical Analysis System version 9.1.3 for Windows (SAS Institute Inc., Cary, NC, USA) was used for statistical analysis.

## Results

### Demographic and breast cancer biological characteristics

Data were collected from a total of 1894 patients in the 63 participating Italian oncology centres. Demographic data are summarized in Table 1. Tumor characteristics and biological classification based on ER, PgR, Ki67 or MIB, HER2 are summarized in Table 2.

**Table 1:** Demographic data of valuable patients

**Demographic characteristics**	**n (%)**
Total	1894 (100)
Median age range (years)	
≥18 − 34	30 (1.6)
≥35 − 49	469 (24.8)
≥50 − 69	984 (51.9)
≥70	411 (21.7)
ECOG^*^ Performance Status	
0	1661 (87.7)
1	179 (9.5)
2	25 (1.3)
3	6 (0.3)
Missing	23 (1.2)
Menopausal status	
Premenopausal	566 (29.9)
Postmenopausal	1307 (69.0)
Missing	21 (1.1)
Second diagnosis of breast cancer	
Yes	86 (4.5)
No	1802 (95.2)
Not reported	6 (0.3)

* ECOG = Eastern Cooperative Oncology Group.

**Table 2:** Tumor characteristics of evaluable patients

**Disease characteristic (n = 1894)**	**Total, n (%)**	**CT, n (%)**	**No CT, n (%)**
Tumor dimensions (pT)			
· pT1mic-pT1a-pT1b	422 (22.3%)	132 (31.3%)	290 (68.7%)
· pT1c	846 (44.7%)	515 (60.9%)	331 (39.1%)
· pT2-pT3-pT4b	626 (33.0%)	448 (71.6%)	178 (28.4%)
Axillary lymph node status (pN)			
· pN0	1155 (61.0%)	502 (43.5%)	653 (56.5%)
· pN1	551 (29.1%)	422 (76.6%)	129 (23.4%)
· pN2	119 (6.3%)	106 (89.1%)	13 (10.9%)
· pN3	69 (3.6%)	65 (94.2%)	4 (5.8%)
Estrogen and Progesterone Receptor (ER/PgR)			
· ER or PgR-positive (≥1%)	1619 (85.5%)	829 (51.2%)	790 (48.8%)
· ER and PgR 0%	274 (14.5%)	266 (97.1%)	8 (2.9%)
· Unknown	1 (0.1%)	0 (0.0%)	1 (100%)
Proliferative index (Ki67 or MIB-1)			
· Low (≤20%)	1150 (60.7%)	499 (43.4%)	651 (56.6%)
· High (>20%)	691 (36.5%)	562 (81.3%)	129 (18.7%)
· Unknown	53 (2.8%)	34 (64.2%)	19 (35.8%)
HER2			
· Positive	305 (16.1%)	269 (88.2%)	36 (11.8%)
· Negative	1463 (77.2%)	761 (52.0%)	702 (48.0%)
· Unknown	126 (6.7%)	65 (51.6%)	61 (48.4%)
Biological type			
· Luminal A	923 (48.7%)	352 (38.1%)	571 (61.9%)
· Luminal B	342 (18.1%)	230 (67.3%)	112 (32.7%)
· HER2	305 (16.1%)	269 (88.2%)	36 (11.8%)
· Triple negative	165 (8.7%)	161 (97.6%)	4 (2.4%)
· Unknown	159 (8.4%)	83 (52.2%)	76 (47.8%)

Luminal A: ER or PgR ≥1% and proliferative index ≤20% and HER2-negative.

Luminal B: ER or PgR ≥1% and proliferative index >20% and HER2-negative.

HER2-positive: HER2-positive (IHC 3+, or IHC 2+ with HER2 amplified, HER2 amplified independently from IHC) independently from the values of ER, PgR, proliferative index.

Triple negative: ER and PgR both 0% and HER2 negative independently from the proliferative index.

The median tumor diameter was 1.7 cm (range: 0.1 to 35.0 cm). Overall 22.3% of tumors were ≤1.0 cm in diameter; pT1c was the more frequent breast cancer pT stage: 44.7%. The median number of involved lymph nodes was 1 (range: 0 to 47), 61.0% were pN0 and 9.9% pN2 or pN3.

With respect to tumor biological characteristics, ER was ≥10% in 82.7%, ER ≥1% in 84.9%. When also considering PgR, ER or PgR were ≥1% in 85.5%, ER or PgR ≥10% in 83.5%, ER and PgR both <10% in 16.4%, ER and PgR both 0% in 14.5%.

HER2 was positive in 16.1% of study population, negative in 77.2%, unknown in 6.7%. According to immunohistochemistry, 13.5% were HER2 3+, 11.6% HER2 2+, 20.5% HER2 1+ and 47.8% HER2 0; an amplification test (FISH, SISH, or CISH) was performed in 22.6% cases. 86.8% HER2 2+ were analyzed with amplification test, of which 26.3% HER2 resulted as amplified.

Proliferative index was known in 97.2%. Among these patients, proliferative index was determined with ki67 in 67.2% and with MIB-1 in 32.8%. Ki67 or MIB-1 was ≤20% in 62.5%, from 21% to 30% in 16.3%, >30% in 21.2% cases. Considering different cut-off values, ≤14% in 42.5%, >14% in 57.5%.

### Factors affecting choice of adjuvant chemotherapy and type of chemotherapy

57.8% (n = 1095) of the study population received adjuvant chemotherapy. A summary of adjuvant chemotherapy type and the administration schedules are listed in Table 3. Table 4 presents factors associated with chemotherapy prescription.

**Table 3:** Type and schedules of adjuvant chemotherapy prescribed (n = 1095)

**Type of chemotherapy**	**N (%)**
CMF-like	100 (9.1)
· Anthracyclines alone (without Taxanes)	534 (48.8)
· Anthracyclines plus Taxanes	420 (38.4)
· Taxanes alone (without Anthracyclines)	41 (3.7)
Type of Taxane	
· Docetaxel	359 (32.8)
· Paclitaxel	102 (9.3)
Main chemotherapeutic schedules administered	
· Oral classical CMF 6 cycles	24 (2.2)
· CMF 6 cycles iv day 1 and 8 q28d	53 (4.8)
· CMF 6 cycles iv day 1 q21d	9 (0.8)
· CMF 3–4 cycles iv day 1 and 8 q28d	11 (1.0)
· AC 4 cycles	89 (8.1)
· AC 6 cycles	27 (2.5)
· E_90_C 4 cycles	56 (5.1)
· E_75_C 4 cycles	9 (0.8)
· FE_75_C 6 cycles	131 (12.0)
· FE_90_C 6 cycles	95 (8.7)
· FE_100_C 6 cycles	29 (2.6)
· FAC 6 cycles	8 (0.7)
· Canadian CEF 6 cycles	16 (1.5)
· Epidoxorubicin 4 cycles – CMF 4 cycles	5 (0.5)
· AC 4 cycles – CMF 3 cycles	15 (1.4)
· FEC 3 cycles - Docetaxel 3 cycles	118 (10.8)
· AC/EC 4 cycles - Docetaxel 4 cycles	127 (11.6)
· TAC 6 cycles + G-CSF	29 (2.7)
· AC/EC 4 cycles - Paclitaxel q3wks 4 cycles	39 (3.6)
· FEC/EC 4 cycles - weekly Paclitaxel x 12	43 (3.9)
· A/EP 4 cycles – CMF 4 cycles	7 (0.6)
· ET 6 cycles	12 (1.1)
· TC 4 cycles	19 (1.7)
· TC 6 cycles	8 (0.7)
· Others	116 (10.6)

C = Cyclophosphamide; M = Methotrexate; F = Fluorouracil; A = Doxorubicin; E = Epirubicin; P = Paclitaxel; T = Docetaxel.

**Table 4:** Summary of the results of the multivariate logistic model analysis that evaluated the probability of being treated with adjuvant chemotherapy (n = 1894)

**Variable**	**OR (95%CI)**	**p value**
Age range (years)		<0.0001
≥70	1	
≥50-69	16.3 (10.4-25.5)	
≥35-49	46.8 (27.5-79.5)	
≥18-34	553 (NE*)	
pT		<0.0001
pT1mic/pT1a/pT1b	1	
pT1c	3.3 (2.2-4.8)	
pT2/pT3/pT4	6.5 (4.2-10.2)	
pN		<0.0001
pN0	1	
pN1	7.6 (5.4-10.7)	
pN2	45.9 (17.9-117.9)	
pN3	28.2 (8.1-97.9)	
Tumor grading		<0.0001
G1	1	
G2	2.8 (1.7-4.6)	
G3	7.2 (4.1-12.7)	
Estrogen receptor		<0.0001
Positive	1	
Negative	24.1 (4.9-119.5)	
Biological type		<0.0001
Luminal A	1	
Luminal B	2.1 (1.5-3.1)	
HER2	10.3 (6.1-17.6)	
Triple negative	4.5 (0.6-31.9)	

* CI not calculable due to the small sample size.

Age was a strong determinant for chemotherapy prescription. Overall, there were no differences in the chemotherapy type prescribed according to age, although use of CMF-like regimen was more common among patients aged ≥70 years. While CMF-like therapy was prescribed in 9.1% of the overall population receiving adjuvant chemotherapy, the probability increased to 27.0% in patients aged ≥70 years; however, the difference was not statistically significant due to low numbers.

Menopausal status influenced the decision to use adjuvant chemotherapy: 74.9% of premenopausal and 50.3% of postmenopausal patients received adjuvant chemotherapy, respectively (OR: 2.95, CI95%: 2.37-3.67, p < 0.0001). Premenopausal patients were more often treated with adjuvant anthracyclines, with or without taxanes, than postmenopausal patients (92.7% versus 83.7%: OR = 2.46, CI95% = 1.62-3.75, p < 0.0001). This difference was not significant considering anthracyclines alone: 49.8% versus 48.5%, respectively (p = 0.68), but was significant for anthracyclines plus taxanes: 42.9% versus 35.3%, respectively (OR = 1.38, CI95% = 1.08-1.77, p = 0.011).

Tumor stage at first diagnosis greatly influenced the decision to use adjuvant chemotherapy as shown in Table 4. The pT category influenced the type of chemotherapy administered: the use of CMF-like therapy decreased from 13.6% of pT1mic/pT1a/ pT1b to 9.9% of pT1c and to 6.9% of pT2/pT3/pT4b (pT1mic/pT1a/ pT1b versus pT2/pT3/pT4b: OR = 2.12, CI95% = 1.15-3.94; pT1c versus pT2/pT3/pT4b: OR = 1.48, CI95% = 0.93-2.36, p = 0.047) At the same time anthracyclines plus taxanes increased from 25.8% of pT1mic/pT1a/pT1b to 34.6% of pT1c and to 46.4% of pT2/pT3/pT4b (pT2/pT3/pT4b versus pT1mic/pT1a/pT1b: OR = 2.50, CI95% = 1.62-3.85; pT1c versus pT1mic/pT1a/pT1b: OR = 1.52, CI95% = 0.99-2.34, p < 0.0001). The use of anthracyclines alone decreased from pT1mic/pT1a/pT1b to pT1c (58.3% and 50.9%, respectively) and to pT2/pT3/pT4b (43.5%) (pT1mic/pT1a/pT1b versus pT2/pT3/pT4b: OR = 1.82 CI95% = 1.23-2.69; pT1c versus pT2/pT3/pT4b: OR = 1.34 CI95% = 1.04-1.73; p = 0.005).

A correlation was also seen between pN staging and higher rates of adjuvant chemotherapy prescription. Anthracyclines plus taxanes use increased with pN status: 19.9% of pN0 patients with prescription of adjuvant chemotherapy received anthracyclines plus taxanes, increasing to 45.0% for pN1 patients and 76.0% in pN2/pN3 patients (pN1 versus pN0: OR = 3.29, CI95% = 2.46-4.41; pN2-pN3 versus pN0: OR = 12.75, CI95% = 8.43-19.28; p < 0.0001). At the same time the use of CMF-like regimens decreased with higher pN: 13.3% of pN0 patients compared to 5.6% of lymph-node positive (pN1-pN2-pN3) (OR = 2.61, CI95% = 1.69-4.04, p < 0.0001). The use of anthracyclines alone decreased from 63.7% of pN0, to 44.8% of pN1 and to 14.6% of pN2-pN3 (pN0 versus pN2-pN3: OR = 10.26, CI95% = 6.47-16.28; pN1 versus pN2-pN3: OR = 4.74, CI95% = 2.97-7.54, p < 0.0001).

Combining pathological stage (IA, IB, II, III) with biological characteristics of breast cancer (luminal A, luminal B, HER2, triple negative) (Table 5) we found that decision for prescribing adjuvant chemotherapy is conditioned by both variables. Increasing pathological stage is associated with more administration of adjuvant chemotherapy independently by biological subtype. In particular, in luminal A 28.6% of patients with pT1 and pN0 and 56.2% in stage II received adjuvant chemotherapy. Compared to luminal A disease, luminal B received chemotherapy more frequently; stage IA: 30.3% versus 6.0% (OR = 6.78, 95%CI = 2.64-17.43, p < 0.0001); stage IB: 50.6% versus 22.6 (OR = 3.50, 95%CI = 2.10-5.84, p < 0.0001); stage II: 76.0% versus 56.2% (OR = 2.47, 95%CI = 1.65-3.71, p < 0.0001); stage III: 90.2% versus 81.0% (p = 0.16).

**Table 5:** Prescription of adjuvant chemotherapy according to biological type and stage. Chi square test was significant (p < 0.0001) for each biological subtype

**Stage**	**Luminal A**		**Luminal B**		**HER2**		**Triple Negative**	
	**CT yes, N (%)**	**CT no, N (%)**	**CT yes, N (%)**	**CT no, N (%)**	**CT yes, N (%)**	**CT no, N (%)**	**CT yes, N (%)**	**CT no, N(%)**
IA*	12 (6.0%)	187 (94.0%)	10 (30.3%)	23 (69.7%)	25 (64.1%)	14 (35.9%)	17 (81.0%)	4 (19.0%)
IB**	59 (22.6%)	202 (77.4%)	44 (50.6%)	43 (49.4%)	77 (88.5%)	10 (11.5%)	56 (100%)	0 (0%)
II***	213 (56.2%)	166 (43.8%)	130 (76.0%)	41 (24.0%)	122 (93.1%)	9 (6.9%)	75 (100%)	0 (0%)
III****	68 (81.0%)	16 (19.0%)	46 (90.2%)	5 (9.8%)	45 (93.8%)	3 (6.3%)	13 (100%)	0 (0%)
Total	352 (38.1%)	571 (61.9%)	230 (67.3%)	112 (32.7%)	269 (88.2%)	36 (11.8%)	161 (97.6%)	4 (2.4%)

* Stage IA: pT1mic/pT1a/pT1b and pN0 ** Stage IB: pT1c and pN0 *** Stage II: pT1 and pN1, pT2/pT3 and pN0, pT2 and pN1 **** Stage III: all pN2, all pN3, all pT4, pT3 and pN1.

Patients with ER 0% and PgR 0% received adjuvant chemotherapy more often compared to patients with ER or PgR ≥1% (97.1% versus 52.1%, respectively; OR = 31.69, CI95% = 15.58-64.44, p < 0.0001). There were no differences in the type of adjuvant chemotherapy with respect to ER/PgR status: CMF-like therapy was prescribed in 9.8% and 8.9% in ER and PgR 0% and ER or PgR ≥1% (p = 0.67), respectively; anthracyclines without taxanes: 50.4% and 48.3%, respectively (p = 0.55); anthracyclines plus taxanes: 36.1% and 39.1%, respectively (p = 0.38); taxanes alone: 3.8% and 3.7%, respectively (p = 0.99). Between triple negative, 97.6% was treated with adjuvant chemotherapy: 81.0% stage IA compared to 100% of stage IB, II, III (Table 5).

64.1% of patients with HER2-positive disease received adjuvant chemotherapy with a tumor diameter until 1 cm of diameter (stage IA). In HER2 disease stage IB + II + III 90.7% of HER2-positive breast cancer patients received adjuvant chemotherapy (HER2 stage IB + II + III versus HER2 stage IA: OR = 6.21, CI95% = 2.83-13.64, p < 0.0001). In particular, in stage IA HER2-positive disease, 85.7% of hormonal receptors negative versus 52.0% of hormonal receptor positive received adjuvant chemotherapy.

HER2 status determined the choice of a regimen containing anthracyclines (with or without taxanes): 94.4% HER2-positive cases versus 85.0% HER2-negative (OR = 2.98, CI95% = 1.71-5.21, p = 0.0001). This data must be particularly applied for regimens with anthracyclines plus taxanes (43.5% versus 36.6%, respectively: OR = 1.33, CI95% = 1.01-1.76, p = 0.048) but not for regimens containing anthracyclines alone without taxanes (50.9% versus 48.4%, respectively, p = 0.47).

Analyzing HER2 as compared to other biological subtype (Table 6), prescription of CMF was constant between luminal A (11.4%), luminal B (7.8%) and triple-negative (10.6%), while it was little decreasing in HER2-positive disease (4.1%) (luminal A + B + triple negative versus HER2: OR 2.63 CI95%: 1.38-5.04, p = 0.004). At the same time there is an increasing use of anthracyclines and taxanes in HER2-positive disease (43.5%) versus luminal A (36.6%), luminal B (37.0%), triple negative (36.6%). This difference was borderline significant (HER2+ versus luminal A + B + triple negative; p = 0.06).

**Table 6:** Type of chemotherapy prescribed according to biological type

**Type of chemotherapy**	**Luminal A**	**Luminal B**	**HER2**	**Triple Negative**
CMF	40 (11.4%)	18 (7.8%)	11 (4.1%)	17 (10.6%)
Anthracyclines	169 (48.0%)	116 (50.4%)	137 (50.9%)	76 (47.2%)
Anthracyclines-Taxanes	129 (36.6%)	85 (37.0%)	117 (43.5%)	59 (36.6%)
Taxanes	14 (4.0%)	11 (4.8%)	4 (1.5%)	9 (5.6%)
Total	352 (100%)	230 (100%)	269 (100%)	161 (100%)

Combining pathological stage with biological characteristics (Table 7) we noted that administration of anthracyclines and taxanes increased with increasing pathological stage independently from biological subtype. At the same time the administration of anthracyclines alone decreases with increasing pathological stage.

**Table 7:** Prescription of the type of chemotherapy based on pathological stage and biology

**Stage**	**CMF-like**	**Anthracyclines**	**Anthracyclines + Taxanes**	**Taxanes**
Luminal A				
IA*	3 (25.0%)	8 (66.7%)	0 (0%)	1 (8.3%)
IB**	16 (27.1%)	36 (61.0%)	7 (11.9%)	0 (0%)
II***	16 (7.5%)	113 (53.1%)	75 (35.2%)	9 (4.2%)
III****	5 (7.4%)	12 (17.6%)	47 (69.1%)	4 (5.9%)
Total	40 (11.4%)	169 (48.0%)	129 (36.6%)	14 (4.0%)
Luminal B				
A*	1 (10.0%)	9 (66.7%)	0 (0%)	0 (0%)
IB**	7 (15.9%)	31 (61.0%)	7 (11.4%)	1 (2.3%)
II***	10 (7.7%)	67 (53.1%)	44 (33.8%)	9 (6.9%)
III****	0 (0%)	9 (17.6%)	36 (78.3%)	1 (2.2%)
Total	18 (7.8%)	116 (50.4%)	85 (37.0%)	11 (4.8%)
HER2				
IA*	0 (0%)	22 (88.0%)	2 (8.0%)	1 (4.0%)
IB**	4 (5.2%)	54 (70.1%)	18 (23.4%)	1 (1.3%)
II***	4 (3.3%)	55 (45.1%)	61 (50.0%)	2 (1.6%)
III****	3 (6.7%)	6 (13.3%)	36 (80.0%)	0 (0%)
Total	11 (4.1%)	137 (50.9%)	117 (43.5%)	4 (1.5%)
Triple negative				
IA*	4 (23.5%)	8 (47.1%)	4 (23.5%)	1 (5.9%)
IB**	8 (14.3%)	30 (53.6%)	14 (25.0%)	4 (7.1%)
II***	3 (4.0%)	38 (50.7%)	32 (42.7%)	2 (2.7%)
III****	2 (15.4%)	0 (0%)	9 (69.2%)	2 (15.4%)
Total	17 (10.6%)	76 (47.2%)	59 (36.6%)	9 (5.6%)

## Discussions

The results of this observational study, conducted throughout oncologic centres of Northern, Central and Southern Italy, is representative of Italy with respect to the choice of adjuvant chemotherapy for breast cancer.

The first important consideration is about demography and biology of breast cancer of this population study. In fact, most patients (85.5%) had ER or PgR-positive (≥1%) breast cancer, while only 14.5% had both ER and PgR 0%. Triple negative cases were 8.7%, 16.1% HER2-positive independently from hormonal receptors status. These results are probably influenced by a high proportion of postmenopausal patients (nearly 70%) and by those aged ≥70 years (21.7% of the population study). In the previous reported observational study conducted by Cazzaniga et al. between 2000 and 2004 [17], elderly patients were 18.5%, despite the biological characteristics being similar to this study.

The second important consideration is about stage. In the current study there is a lower incidence of high-risk nodal disease (pN2-pN3), which decreased from 17.1% of NORA to 9.9% of NEMESI study (p = 0.0001). This is probably due to more extensive use of breast cancer screening in recent years in Italy. Probably, for the same reason, pT1 increased from 59.7% of patients in NORA to 67.0% of NEMESI (p < 0.0001).

These demographical, pathological and biological characteristics seem to be in contrast with the high rate of adjuvant chemotherapy prescription. 57.8% of the population study received adjuvant chemotherapy. Population study was composed by 85% of endocrine responsive breast cancers, 84% of HER2 negative, 61% of node-negative and 2 of 3 cases with tumor diameter inferior to 2 cm, 22.3% ≤1 cm. International guidelines recommend the use of adjuvant chemotherapy on the basis of all prognostic and predictive parameters but in general it is not recommended for patients with pT1, pN0, ER-positive, low proliferation index, HER2 negative breast cancer. We noted that, overall, 38.1% of luminal A population was treated with adjuvant chemotherapy: 6% in stage IA, 22.6% in stage IB, 56.2% in stage II, 81.0% in stage III. It means that 33.8% of luminal A with low (pT1 pN0) or intermediate recurrence risk (pT2/pT3 pN0 or pN1) was treated with adjuvant chemotherapy. 25% of entire study population receiving adjuvant chemotherapy was luminal A with low or intermediate risk recurrence. This fact underlines an overtreatment and over prescription with adjuvant chemotherapy. In fact, luminal A is recognized as the less sensitive breast cancer subtype to adjuvant chemotherapy. The cut-off for luminal A was set to a proliferative index ≤20%. As indicated by Cheang et al., ki67 of 14% is considered the proliferation index able to distinguish luminal A versus luminal B [18]. Modifying the cut-off value of proliferative index from 20% to 14% in order to distinguish between luminal A versus luminal B we obtained similar data: 29.5% of luminal A with stage IA, IB, II was treated with adjuvant chemotherapy and OR for the use of adjuvant chemotherapy in luminal B versus luminal A was 1.95 (OR = 1.40-2.72) similar to that reported in Table 4.

NEMESI study identified tumor and patient characteristics that were important determinants in the decision to prescribe a chemotherapy regimen and the choice of chemotherapy type. Whereas adjuvant chemotherapy prescription by Italian oncologists is influenced by prognostic and predictive parameters, type of adjuvant chemotherapy is significantly influenced by age, menopausal status and above all by the risk of recurrence (pT and pN), but not by biological predictive factors. In fact, prescription of CMF-like regimen decreases with increasing pT and pN in favor of the use of anthracyclines plus taxanes. In particular, as previously reported, considering small tumors pT1a and pT1b compared to larger tumors, CMF-like prescription decreases from 14.2% to 9.9% of pT1c and 6.9% of pT2, pT3, pT4b [19]. More recurrence risk is, more is the use of the most effective regimen anthracyclines plus taxanes. The choice for anthracyclines plus taxanes should be considered not on the basis of pT and pN but on the basis of sensitiveness to chemotherapy. As indicated by St. Gallen guidelines, luminal B, HER2 and triple negative should receive both anthracyclines and taxanes, which, on the contrary, are treated with this regimen in only 37.0%, 43.5% and 36.6% of cases, respectively.

Another important issue is about older age and prescription of the type of adjuvant chemotherapy. In fact, administration of CMF-like regimen was more frequently used for women aged ≥70 (27.0%) than for women under 70 (9.1%) and, as previously reported, this percentage increased until 50% considering only pT1a and pT1b tumors [19]. This choice is in contrast with data from randomized trials conducted in elderly women in whom CMF-like was demonstrated to be more toxic and less feasible compared to anthracyclines, like AC regimen [20].

On multivariate analysis, the only biological parameter little influencing the choice of chemotherapy type is HER2. In HER2-positive breast cancer patients, anthracyclines use increased; this is probably also influenced by the results of a meta-analysis by Gennari et al. [21]. There was an overall increase in anthracyclines use in HER2-positive versus HER2-negative patients (absolute increase: 9.4%); however, while anthracyclines alone were prescribed equally between HER2-positive and HER2-negative patients, there was higher use of anthracyclines plus taxanes in HER2-positive versus HER2-negative patients (absolute increase: 6.9%). This data is also consistent with a meta-analysis conducted by De Laurentiis et al. [22] which demonstrated that chemotherapy containing anthracyclines plus taxanes is more effective than anthracyclines alone in HER2-positive breast cancer.

Finally, patients treated with chemotherapy included schedules with CMF-like regimen or AC/EC for four cycles. Recently, EBCTCG demonstrated that these regimens remain suboptimal schedules in terms of efficacy. We know from EBCTCG metanalysis that more effective chemotherapy regimens included anthracyclines with doxorubicin with a dose of at least 60 mg/m^2^ per cycle or epirubicin with a dose of at least 90 mg/m^2^ per cycle with cumulative dose of more than 240 mg/m^2^ and 360 mg/m^2^, respectively, or regimens including anthracyclines plus taxanes [23]. Overall, 25.3% of population study was treated with CMF-like or AC/EC four cycles and 12% with FEC for 6 cycles but with suboptimal epirubicin dose per cycle (75 mg/m^2^). These data were confirmed also for particular sensitive disease to chemotherapy, HER2-positive and triple negative, in which suboptimal chemotherapy with CMF-like or AC/EC for 4 cycles was prescribed in 24.2% and 22.2%, respectively. FEC 6 cycles with epirubicin 75 mg/m^2^ was adopted in 12.3% and 11.8%, respectively.

## Conclusions

This observational study showed a possible 25% of over prescription of adjuvant chemotherapy for early breast cancer, particularly referred to luminal A biological subtype. Prognostic parameters (pT and pN) and only marginally HER2 were the principal determinants for the prescription of the type of chemotherapy, with the use of more effective regimen (anthracyclines and taxanes) with increasing risk recurrence and HER2-positive. Suboptimal chemotherapy regimens (CMF-like or AC/E or FEC with suboptimal epirubicin dose) were adopted in at least one third of highly chemosensitive disease (HER2-positve and triple negative).

## Competing interest

The authors have no conflicts of interest that are directly relevant to the content of this article, including any financial, personal or other relationship with other people or organizations within that could inappropriately influence the results of this work. Davide Dondi is a full-time employee of Sanofi-Aventis Italy. The manuscript was prepared independently from Sanofi-Aventis Italy.

## Authors’ contributions

Matteo Clavarezza was responsible for manuscript writing. All authors read and approved the final paper.

## Pre-publication history

The pre-publication history for this paper can be accessed here:

http://www.biomedcentral.com/1471-2407/12/216/prepub
